# The Emergence of Rod-Cone Cellular Interaction

**DOI:** 10.3389/fgene.2022.900849

**Published:** 2022-08-09

**Authors:** Najate Aït-Ali, Thierry Léveillard

**Affiliations:** Department of Genetics, CNRS, INSERM, Institut de la Vision, Sorbonne Université, Paris, France

**Keywords:** photoreceptors, glycolysis, thioredoxin, basigin, cell-cell communication, evolution

## Abstract

We studied the origin of rod-derived cone viability factor (RdCVF) during evolution. In mammals, the nucleoredoxin-like 1 gene (*NXNL1*) produces a truncated thioredoxin-like protein, RdCVF, by intron retention in rod photoreceptors of the retina. This protein prevents the secondary cone degeneration in animal models of rod-cone degeneration. Extracellular RdCVF binds to a complex at the surface of the cones, composed of the basigin-1, a photoreceptor specific alternative splicing product of the basigin gene, and GLUT1, the glucose transporter. RdCVF accelerates glucose uptake allosterically. Glucose is either metabolized by aerobic glycolysis to sustain cone outer segment renewal or by the pentose phosphate pathway to support redox power to the thioredoxin RdCVFL. RdCVF signaling predates the appearance of the eye and evolved through two alternative splicing events. RdCVF signaling is observed first in hydra where it regulates an unknown signaling. A scallop RdCVF protein is produced by ciliated photoreceptors of the retina and binds its receptor, BSG1, the first occurrence of RdCVF/BSG1 signaling. In the lamprey, RdCVF metabolic signaling between rod and cones is fully operational. In the mouse, the production of BSG1 is regulated through alternative splicing. This signaling was extended to other regions of the brain, via its paralogue *NXNL2*.

## Introduction

With only 2% of human body mass, the brain of an adult human uses about 15–20% of the body’s total glucose-derived energy (Bauernfeind and Babbitt, 2020). The brain depends upon glucose as its main source of energy (Mergenthaler et al., 2013). In the adult brain, it is the neurons that have the highest energy demand, requiring continuous delivery of glucose from blood. In all multicellular organisms, ATP, the major energy source for all cells in the body, is predominantly supplied by metabolism of glucose by glycolysis and oxidative phosphorylation (Bauernfeind et al., 2014). In addition to being used as a source of energy, glucose can also be metabolized through anaerobic pathways to create biomolecules that are essential for neurons. Aerobic glycolysis occurs when glucose is incompletely metabolized to carbon dioxide and water, so that the intermediate molecules are used in anabolism pathways, as cancer cells do (Vander Heiden et al., 2009). During postnatal brain development, aerobic glycolysis is required for axonal elongation, myelination, and synaptogenesis. In adulthood, proteins, lipids, and amino acids are continuously synthesized to replace constituent molecules and to support neuronal plasticity by an increase of dendritic spines and synapses. Because human brain development is prolonged compared to nonhuman primates (NHP), humans require elevated levels of glucose for a longer duration than NHP. Several observations suggest that humans have benefited from molecules that have evolved during the primate lineage to allow brain to use energy more efficiently. Genomic data show that glucose metabolism has been a target of natural selection in primate evolution (Bauernfeind and Babbitt, 2014). In the genomes of humans and chimpanzees, the rates of sequence evolution in promoter regions of genes supporting glucose metabolism, as hexokinase 1, evolved more quickly than its associated coding region, a sign of positive selection. In addition, the upregulation of metabolic genes in the adult human neocortex suggests that, for the same brain weight unit, the human brain is more metabolically active than the brains of other adult primates.

**FIGURE 1:**
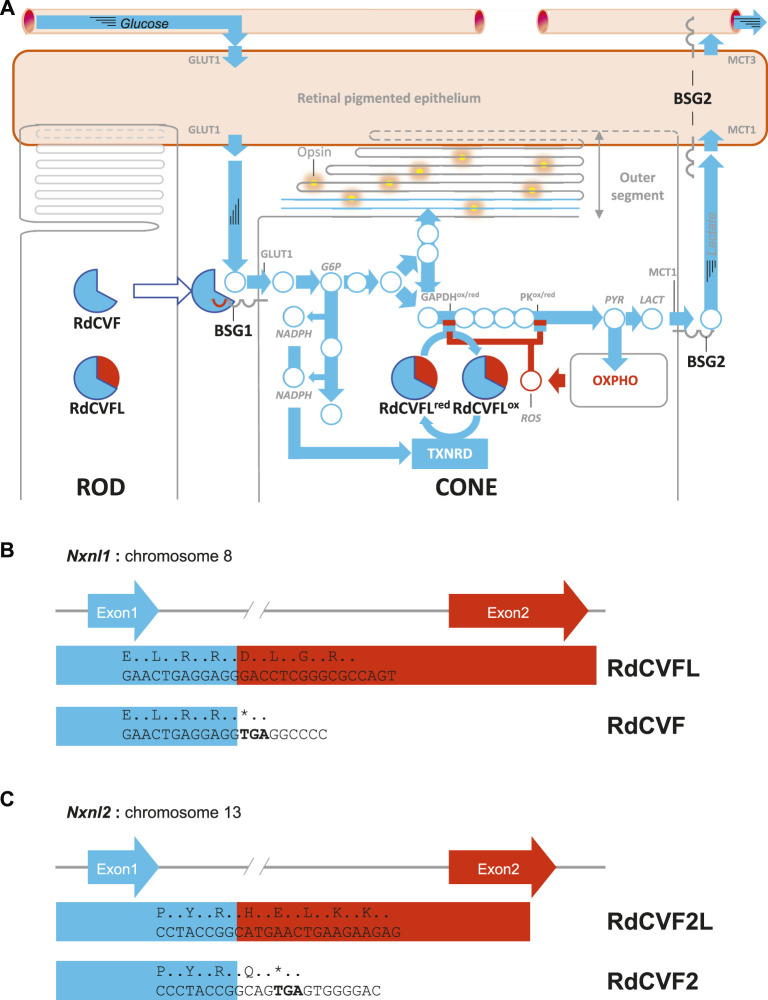
The metabolic and redox signaling of the nucleoredoxin-like genes. **(A)** The cellular interaction between rods and cones in the retina. BSG1: basigin-1, BSG2, basigin-2, G6P: glucose-6-phosphate, GAPDH: glyceraldehyde-3-phosphate dehydrogenase, GLUT1: facilitated glucose transporter SLC2A1, LACT: lactate, MCT1: lactate transporter MCT1, MCT3: lactate transporter MCT3, NADP^+^: oxidized nicotinamide adenine dinucleotide phosphate, NADPH: reduced nicotinamide adenine dinucleotide phosphate, PK: pyruvate kinase, PYR: pyruvate, RdCVF: rod-derived cone viability factor, RdCVFL: rod-derived cone viability factor long, ROS: reactive oxygen species, TXNRD: thioredoxin reductase. **(B)** Schematic representation of the mouse nucleoredoxin-like 1 gene. **(C)** Schematic representation of the mouse nucleoredoxin-like 2 gene.

It is the arteries of the brain that supply oxygenated blood and glucose to neurons and glia of the brain. Neurons are highly demanding of energy, especially upon activation. The brain is supplied by two internal carotid arteries and two vertebral arteries which branches into numerous arterioles (Agarwal and Carare, 2020). The non-fenestrated endothelium of the arterioles constitutes the blood-brain barrier. Neurovascular coupling is a rise in cerebral blood flow occurring in a timely and spatially restricted manner to the activated brain area in order to ensure adequate supply of oxygen and glucose to the activated neurons (Lecrux et al., 2019). Neurovascular coupling is at the basis of brain neuroimaging relying on local changes in blood flow. Hemodynamic-based neuronal activity can be imaged in the whole brain, non-invasively and repeatedly as surrogate of neuronal activity. Positron emission tomography (PET) is used to image cerebral blood flow and also the cerebral metabolic rate for oxygen using ^15^O (Lameka et al., 2016). In parallel, ^18^F-fluorodeoxyglucose (FDG) was developed to quantify brain glucose metabolism by PET (Muzi et al., 2001). Aerobic glycolysis is measured as the molar ratio of oxygen consumption to glucose utilization, the oxygen-glucose index (Vaishnavi et al., 2010). As a glucose derivative, FDG is transported into cells by glucose facilitated transporters (GLUT/SLC2A) and then subsequently phosphorylated by hexokinases into FDG-6-phosphate similarly to glucose is metabolized into glucose-6-phosphate (G6P) (Figure 1A). FDG-6-phosphate and G6P do not exit the cell, so the phosphorylation of FDG is the rate limiting step in the accumulation of PET signal, which undergoes very slow fading during the exam since FDG-6-phosphate is not further metabolized, contrarily to G6P. In rigorous enzymatic terms, quantitative PET images are proportional to the metabolic rate of FDG described by Michaelis-Menten constants for hexokinase 1 (HK1) and hexokinase 2 (HK2). In clinical practice and clinical research, quantitative imaging of FDG is a measurement of glucose uptake, so of the activity of glucose facilitated transporters. Any molecule that accelerates glucose uptake has a profound effect on the whole system.

## Metabolic and Redox Signaling by the Nucleoredoxin-Like 1 Gene

Retinitis pigmentosa (RP), also called rod-cone degeneration is a genetically heterogeneous retinal degenerative disease characterized by the death of rod photoreceptors followed by the progressive loss of function and degeneration of cones, whatever the expression pattern of the mutated gene (Verbakel et al., 2018). RP is the most common form of inherited retinal degeneration, affecting around two million people worldwide (Ferrari et al., 2011). Originally, the identification of a dominant mutation in the gene encoding for rhodopsin, the visual pigment of rods, explained the death of rods but not the secondary degeneration of cones since this gene is not expressed by cones (Dryja et al., 1990). The reason why a mutation in a gene expressed exclusively in rods can lead to loss of function and degeneration of cones was then proposed to results from the loss of non-cell autonomous protective effect of rod-derived cone viability factor (RdCVF), encoded by the nucleoredoxin-like 1 gene (*Nxnl1*) (Leveillard et al., 2004; Leveillard and Sahel, 2010). RdCVF is produced, secreted by rods and binds on the surface of the cone to basigin-1 (BSG1), the latter being in complex with the glucose facilitated transporter, GLUT1 (Ait-Ali et al., 2015) (Figure 1A). This interaction triggers an allosteric elevation of the uptake of glucose by cones. Cones metabolize glucose by glycolysis producing two molecules of pyruvate that are transported into the mitochondria where they are further processed by the Krebs cycle and the respiratory chain to produce ATP as for most neurons (Hayes et al., 2021). Mitochondria generate, as byproducts, radical oxygen species (ROS) because of leakage of the respiratory chain. Cones also convert pyruvate into lactate in the presence of oxygen by the aerobic glycolysis. Lactate is first transported out of the cones by lactate facilitated transporters MCT1 then though the retinal pigmented epithelium (RPE) by MCT1 and MCT3 located on the RPE apical and basal side, respectively (Leveillard et al., 2019). For reasons that remain unexplained today, the process of aerobic glycolysis is required for the diversification of glucose metabolism that allows phospholipid synthesis through the Kennedy pathway, which is needed for the renewal of cone outer segments (Leveillard, 2015). The cones have been shown to be vulnerable to oxidative stress (Komeima et al., 2006). Glucose metabolism by cones is negatively regulated by ROS that inhibit by cysteines oxidation two glycolytic enzymes, glyceraldehyde-3-phosphate dehydrogenase (GAPDH) and pyruvate kinase M2 (PKM2) (Anastasiou et al., 2011; Reisz et al., 2016). The inhibition of the glycolytic flux triggers the metabolism of G6P, upstream in the chain of enzymatic reactions, by the pentose phosphate pathway (Figure 1A). This produces the reduction of NADP^+^ into NADPH, the cofactor of thioredoxin reductases, that reduce thioredoxins, as such as RdCVFL. RdCVFL is expressed by both type of photoreceptors, contrarily to RdCVF that is only expressed by rods (Elachouri et al., 2015; Mei et al., 2016). Since RdCVFL was shown to interact with PKM2 (Fridlich et al., 2009), RdCVFL could reduce the oxidized cysteines of PKM2 and reestablish the glycolytic flux (Camacho et al., 2019). The protection of cones by the two products of the *NXNL1* gene is a therapeutic strategy under development for all genetic forms of RP (Clerin et al., 2020).

## The Origin of Rod-Derived Cone Viability Factor

The mouse *Nxnl1* gene is composed of two coding exons (Figure 1B). Splicing of the unique intron generates a messenger RNA that encodes for the thioredoxin RdCVFL and intron retention, a mRNA encoding for RdCVF. The 109 residues of RdCVF are identical to the N-terminal region of the 217 amino acids long RdCVFL protein, since RdCVF is truncated within the thioredoxin fold. When administrated by gene therapy by an ubiquitous promoter, RdCVFL reduced the retinal concentration of lipid hydroperoxides of a recessive model of RP, the *rd10* mouse, but this does not rescue cone function (Byrne et al., 2015). When administrated by gene therapy through a cone-specific promoter, RdCVFL recues cone function, but not RdCVF (Mei et al., 2016). This apparent discrepancy indicates that the target molecules of the thioredoxin RdCVFL are reduced inside the cones. The absence of benefit for cones of RdCVF when it is expressed under the control of a cone-specific promoter is explained by the fact that cones, that make 3% of photoreceptors in the mouse retina, cannot produce a sufficiently high extracellular concentration of RdCVF to activate BSG1/GLUT1. The combination of the non-cell autonomous action of RdCVF and the cell-autonomous action of RdCVFL was modelled as the metabolic and redox signaling in the retina (Leveillard and Sahel, 2016). In an evolutionary perspective, RdCVFL should precede RdCVF, since the latter has a truncated thioredoxin fold that appeared first (Leveillard and Ait-Ali, 2017). *Nxnl1* is expressed specifically in the retina, but its paralogue, *Nxnl2*, is also involved in the protection of the function of olfactory sensitive neurons and pyramidal neurons of the hippocampus (Jaillard et al., 2012; Jaillard et al., 2021). *Nxnl2* also encodes for a trophic factor and a thioredoxin-like protein by alternative splicing (Figure 1C).

### The Mysterious Role of Rod-Derived Cone Viability Factor in the Eyeless Hydra

**FIGURE 2:**
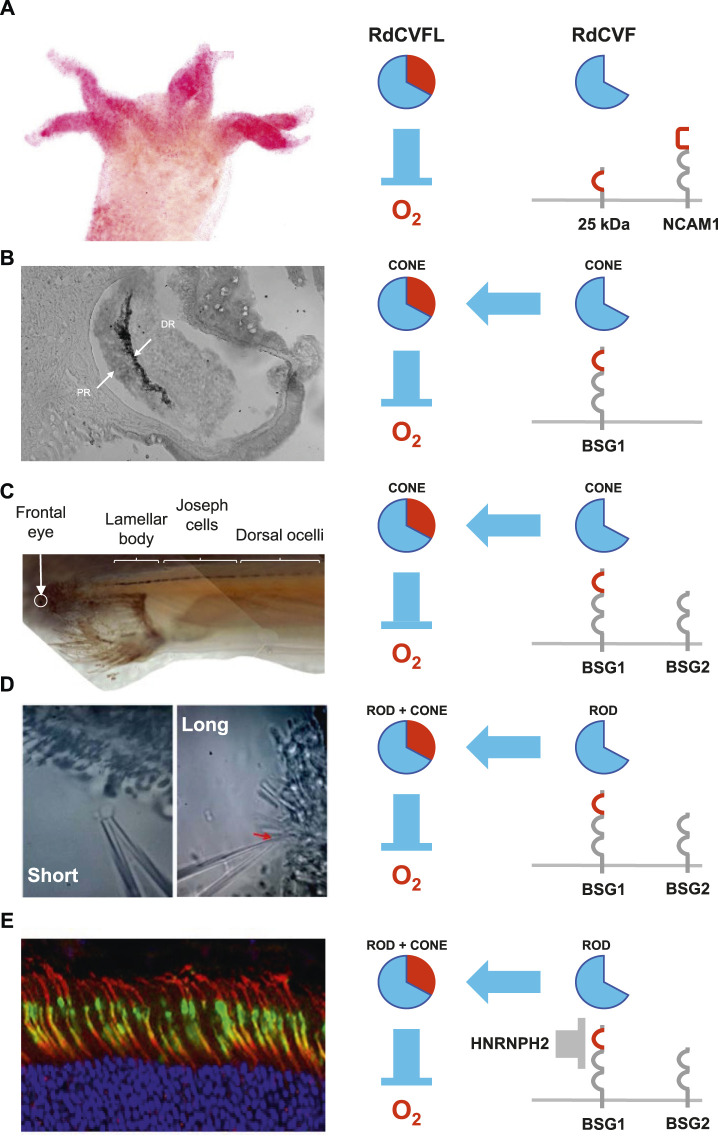
Evolution of the of the nucleoredoxin-like 1 gene. **(A)** *Hydra vulgaris*. **(B)** *Patinopecten yessoensis* (scallop). DR: distal retina. PR. **(C)** *Branchiostoma lanceolatum* (amphioxus). **(D)** *Petromyzon marinus* (lamprey). **(E)** *Mus musculus* (mouse). HNRNPH2: heterogeneous nuclear ribonucleoprotein H2.

We identified by genome mining *NXNL* genes at the basis of the animal kingdom, in sponge and hydra. In *Hydra vulgaris*, two tandemly repeated *NXNL* genes encode for ubiquitously expressed and active RdCVFL thioredoxins that protect the organism against the damages caused by ROS (Ait-Ali et al., 2022). Surprisingly, both *NXNLa* and *NXNLb* also encode for truncated thioredoxins, similar to mammalian RdCVF. *Hydra Vulgaris* has no eye but possesses photosensitive cells located on its tentacles (Passano and McCullough, 1962; Guertin and Kass-Simon, 2015). These photosensitive neurons share with vertebrates the use of the mode of signal transduction of ciliary photoreceptors, as in human (Plachetzki et al., 2012). The most abundant of these truncated proteins, RdCVFa, is expressed specifically by cells of the tentacles suggesting that those cells express a ligand of a cell-surface receptor, what was confirmed by far-western blotting (Figure 2A). The neural-cell-adhesion-molecule-like-1 (NCAM1), the protein in the hydra proteome most closely resembling to BSG1 does not interact with RdCVFa, most probably because its third and N-terminal domain is not homologous to the immunoglobulin domain (Ig) zero of BSG1 that is required for the interaction with RdCVF (Ait-Ali et al., 2015). RdCVFa interacts with a membrane protein of 25 kDa which could consist of an N-terminal Ig0 and a transmembrane domain. The function of this ligand receptor interaction is an open question. Since the interaction of BSG2 with MCT1, and most likely BSG1 with GLUT1, require only the transmembrane domain of BSG1, we can speculate that RdCVFa mediates a metabolic signal. In that respect, *Cnidarians*, as *Hydra vulgaris*, invest a large fraction of their energy in the maintenance of their cnidocyst repertoire, which has to be constantly renewed, like the outer segments of photoreceptors (Beckmann and Ozbek, 2012). Contrarily to RdCVFa, RdCVFb does not interact with the 25 kDa receptor and sequence analysis points to a candidate difference (E > K) that would explain this finding. Nevertheless, our analysis cannot discriminate between two scenarios: either a loss of function of RdCVFb or a gain of function of RdCVFa. In the latter one, RdCVFa would be the first functional truncated thioredoxin appearing (*terminus ad quem*) during evolution. Among the five introns of the *NXNLa* and *NXNLb* genes only intron four is retained, producing RdCVF-like proteins. Introns four have no sequence divergences with the non-retained introns so the genetic mechanism used by *Hydra vulgaris* to produce RdCVF is another yet unresolved mystery. The take home message is that RdCVF signaling predate the appearance of the eye during evolution (Picciani et al., 2021).

### The Role of Rod-Derived Cone Viability Factor for Ciliated Photoreceptors of Scallop

Scallop is a mollusc with up to 200 fully functional eyes. Molluscs, but not *Cnidarians*, are bilaterians and appeared after *Hydra vulgaris* during evolution. They belong to the *Protostomians* that bring together molluscs and arthropods, among which the insects with their rhabdomeric (microvillar) photoreceptors. Those are quite distinct of ciliated photoreceptors, both in terms of morphology and phototransduction. We have reported, as others, that genomes of insects, such as *Drosophila melanogaster* does not contain any *NXNL* genes (Chalmel et al., 2007; Funato and Miki, 2007). The unique scallop *NXNL* gene of *Patinopecten yessoensis* has three coding exons with intron two retention that produces of a RdCVF protein as in *Hydra vulgaris*. This RdCVF protein interacts with its cell surface receptor, a neuroplastin-like protein of the scallop (Ait-Ali et al., 2022). The scallop eye also expresses a RdCVFL protein, most likely an active thioredoxin. At this stage of evolution, the metabolic and redox signaling system was certainly operational (Figure 2B). The peculiarity of the scallop eye is the presence of both ciliary and rhabdomeric photoreceptors (Kojima et al., 1997). The ciliary and rhabdomeric photoreceptors are arranged in two separated retinas, a distal and a proximal retina (Palmer et al., 2017). The two products of the *NXNL* gene are exclusively expressed by the distal retina. This indicates that the gene network that regulates the expression of *NXNL* is functional only in ciliated photoreceptors that renew their outer segments, contrarily to rhabdomeric photoreceptors. The role of the *NXNL* gene is specifically linked to the function of ciliary photoreceptors.

### The First Occurrence of BSG2, the Chaperon of Lactate Transporter, in Amphioxus

Amphioxus, a model of early vertebrate evolution, possesses two types of photoreceptors aligned along its body axis. Amphioxus has ciliary photoreceptors in the head (frontal eye and lamellar body) and rhabdomeric photoreceptors further downstream of its body axis (Joseph cells and dorsal ocelli have) (Lamb, 2013). We identified four *NXNL* genes, arbitrarily named *NXNLa*-*NXNLd* in the *Branchiostoma lanceolatum* genome, each with two exons (Ait-Ali et al., 2022). One of them, *NXNLa*, is expressed in the part of the body containing ciliary photoreceptors, while the three others are expressed at low level in all body parts. The unique intron of *NXNLa* is partially retained leading to the expression of RdCVFa and RdCVFLa proteins. The RdCVFa receptor, BSG1 is widely distributed in the body parts containing ciliary and rhabdomeric photoreceptors and we assume that RdCVFa binds to BSG1. The gene encoding BSG1 also encodes for BSG2 by alternative splicing, a mRNA that is absent in the scallop eye (Figure 2C). This observation supports that the RdCVF receptor (BSG1) precedes the chaperon of the lactate transporter (BSG2) during evolution, a scenario compatible with our hypothetical model of *NXNL1* evolution based on the selection pressure on glucose uptake (Leveillard and Ait-Ali, 2017). We consider here that BSG1 forms a complex with GLUT1, and that RdCVFa increases glucose uptake and aerobic glycolysis, what remains to be demonstrated. BSG2 became an essential element of this metabolic signaling by addressing the lactate transporter MCT1 to the cell membrane (Deora et al., 2005). BSG2 participates to the transport of lactate through the retinal pigment cell, via MCT1 and MCT3 (Vopalensky et al., 2012) (Figure 1A).

### The Metabolic and Redox Signaling of the Nucleoredoxin-Like 1 in the Lamprey Retina

Lamprey represents the most ancient group of vertebrates existing for over 360 million years. It is at this point during evolution when rod evolved from cone, even before the emergence of rod outer segment ultrastructure (Morshedian and Fain, 2015). The evolution of a rod with higher sensitivity may have allowed early vertebrates to inhabit deeper waters. But since cones have elevated energy requirement, this event has reduced the energy cost of the retina and permitted cephalization (Feinberg and Mallatt, 2013; Ingram et al., 2020). The unique *NXNL* gene of the lamprey genome of *Petromyzon marinus* is an orthologue of vertebrate *NXNL1* genes, with an unique intron that is partially retained by rod but not cone photoreceptors (Ait-Ali et al., 2022). Rods produce RdCVF and RdCVFL proteins and cones only RdCVFL, as in the mouse (Mei et al., 2016). This RdCVF protein binds to BSG1 of the lamprey. RdCVF, RdCVFL and BSG1 expression is restricted to the retina, while BSG2 is expressed by the RPE and other tissues (Figure 2D). This timing agrees with the emergence of a metabolic signaling between rods and cones, synchronized to the separation of ancestral cones into rods and cones. So, contrarily to what we could expected, it is not the dominance of rods that outnumber cones in mammalian retina, a results from a nocturnal bottleneck (Borges et al., 2018; Brownstein, 2019) that signs the emergence of rod to cone metabolic interaction since in it is already in place in a species with equivalent number of rods and cones. A competition between rods and cones for a survival signal was proposed by our mathematical modelling of photoreceptor interaction (Camacho et al., 2016). In this model, rods have a metabolic advantage over cones that they compensate by providing a signal that accelerate glucose uptake by cones. Considering the importance of color vision in animal life, natural selection has made rods use of a compensatory mechanism to preserve color vision by feeding cones in an “altruist manner” (Perini et al., 2009; Leveillard et al., 2015). The nature of this hypothetic metabolic advantage is presently unknown, but the metabolic and redox signaling of the *NXNL1* gene dates from that time.

### The Regulation of Expression of BSG2, the Chaperon of Lactate Transporters, by Mouse Retinal Pigment Epithelium

RdCVF secreted by rods binds to the BSG1/GLUT1 complex and accelerates glucose uptake, but BSG1 is also able to interact with the lactate transporter MCT1 (Ait-Ali et al., 2015). In that configuration, the expression of BSG1 by the RPE could potentially modify the kinetics of glucose transport by GLUT1 on the apical site (toward photoreceptors) of the RPE and, doing so, increase the supply of glucose to the outer retina (Figure 1A). Nevertheless, the direction of glucose transport by the facilitated transporter GLUT1 is governed by the difference of glucose concentration between the cytosol of the RPE and the extracellular space of photoreceptors. Glucose is transported from the choroid through the RPE to photoreceptors for their metabolic needs. GLUT1 in the apical side of the RPE has an inverted polarity of glucose transport as compared to photoreceptors, including cones, in this ecosystem (Kanow et al., 2017). We studied the splicing of the *Bsg* gene in the retina and the RPE and concluded that the expression of BSG1 is repressed in the RPE (Ait-Ali et al., 2022). Out of two transgenic mice constructed to inactivate specifically the expression of BSG1, one of them leads accidentally to the expression of BSG1 messenger RNA by the RPE. Since this mRNA contains an in-frame stop codon, BSG1 protein is not expressed leading to photoreceptors loss of function similar to what was observed for the *Bsg* ^−/−^ mouse (Philp et al., 2003). GLUT1 does not require the chaperone activity of BSG2, so that the retinal phenotype results from a deficit in lactate transport.

A bioinformatic analysis indicates that the stop codon introduced in the *Bsg* locus leads to the loss of RNA binding elements of several splicing factors. The comparison of the expression of these splicing factors by the RPE and the outer retina indicates that HNRNPH2 is the splicing inhibitor that represses, in the RPE, the insertion of the exon encoding Ig0 of BSG1 (Figure 2E). We do not know if this splicing inhibition is also taking place in the RPE of the lamprey. As stated above, during evolution BSG1 preceded BSG2. The primary function of BSG1 is that of receptor of RdCVF and probably a allosteric activator of glucose uptake via a facilitated glucose transporter, an orthologous of GLUT1 (Brooks and Storey, 1997). Considering the direction of the metabolic flux in aerobic glycolysis (Figure 1A), the transport of glucose is upstream of that of lactate. In a very simplified picture, the positive effect of RdCVF on glucose uptake via BSG1 precedes the effect of BSG2 on lactate transport. GLUT1 and MCT1 belong the solute carrier family and share 17% identity in human proteome. It is conceivable the gene encoding originally for the RdCVF receptor became recruited to encode by alternative splicing for the chaperon of the lactate transporters. This is speculative but the double function of the basigin in regard to RdCVF metabolic signaling is troubling (Klipfel et al., 2021).

### The Metabolic and Redox Signaling of a Nucleoredoxin-Like Gene Outside the Eye

**FIGURE 3:**
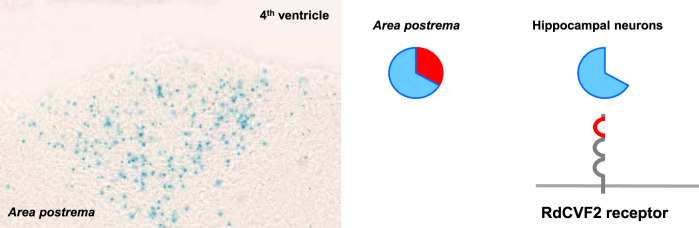
Metabolic and redox signaling of the nucleoredoxin-like two gene in the brain. Cells of the *area postrema* expressing the *Nxnl2* gene secret RdCVF2 that binds to its cell surface receptor on hippocampal neurons.

In the retina, we have shown that *NXNL2*, the paralogue of *NXNL1*, also encodes for a trophic factor, RdCVF2, and a thioredoxin-like protein, RdCVF2L (Chalmel et al., 2007; Jaillard et al., 2012). In addition, the products of the *Nxnl2* gene of the mouse are expressed by olfactory sensitive neurons and by a subset of cells of the *area postrema* (Jaillard et al., 2012; Jaillard et al., 2021). In the mouse, the inactivation of the *Nxnl2* gene results in a complex syndrome with deficits in anxiety, pain sensitivity, coordination and memory. By its juxtaposition the fourth ventricle, a signal generated in the *area postrema* could theoretically circulate in the cerebrospinal fluid to reach neurons in other brain areas, such as the hippocampus (Figure 3). The duplication of the *NXNL1* gene in the descent of the lamprey gave rise to two orthologous genes, *NXNL1* and *NXNL2*, in most fish genomes (Chalmel et al., 2007; Ait-Ali et al., 2022). *NXNL1* and *NXNL2* are expressed by rods of the zebrafish retina (Sun et al., 2018). Zebrafish *NXNL2* is also expressed by the olfactory placode that gives rise to the olfactory epithelium in the adult fish (https://zfin.org/), as what we reported for the mouse (Jaillard et al., 2012). Vision and olfaction are senses that permitted for the first time the emergence of complex behaviors during animal evolution (Feinberg and Mallatt, 2016). Later, interneurons added complexity to the nervous system by increasing the information processing centers to produce a cognitive map of the environment. Since the brain require high level of glucose, it is not surprising that the allosteric activation of glucose uptake by RdCVF in the retina was used, via the RdCVF2 protein, for olfaction and memory since innovation during evolution relies most of the time on preexisting functional units (Jacob, 1977). In the brain, the reduction of glucose metabolism, due to the lack of RdCVF2, would lead to a reduction of the redox power of the enzyme RdCVF2L (Leveillard and Sahel, 2017). In agreement with the model of metabolic and redox signaling, we found aggregated TAU protein, that is induced by oxidation while aging (Fridlich et al., 2009), in the brain of aged *Nxnl2* ^−/−^ mice (Jaillard et al., 2021). TAU aggregation is a penultimate step in the formation of neurofibrillary tangles found in the brain of patients suffering of Alzheimer’s disease.

## Limitations and Future Directions

Our analysis of the emergence of RdCVF signaling stands on common ancestry and of descent with modifications, the foundation of evolutionary theory and also on punctuated equilibrium, a model of evolution with extended periods of evolutionary stasis are punctuated by rapid transitions between states (Bakhtin et al., 2021; Hancock et al., 2021). But since past evolution cannot be experimentally reproduced (Kawecki et al., 2012), our interpretation is of course based on a scenario that optimally fits with our observations.

We plan to identify the cell surface receptor of RdCVFa in *Hydra vulgaris*, a necessary step in the understanding of the original role of this signaling prior to the appearance of the eye during evolution. We will also search for molecular clues that can validate the hypothetic metabolic advantage of rods over cones in the vertebrate retina.

## Conclusion

Our phylogenetic analysis of the *NXNL* genes demonstrates that the function of the truncated thioredoxin-like protein RdCVF, whatever strange this protein is for structural biology, was conserved and recruited during evolution to function in other intercellular communications outside the eye (Table 1). We believe the reason behind this evolutionary success is the capacity of RdCVF to accelerate glucose uptake allosterically.

**TABLE 1:** Summary table.

Species	Gene	RdCVFL	RdCVF	BSG1	BSG2
*Hydra vulgaris*	*NXNLa*	Whole body	Tentacles	Not found, but binding of a unidentified cell surface receptor	Not found
	*NXNLb*	Expression very low	Expression very low		
*Patinopecten yessoensis* (Scallop)	*NXNL*	Distal retina (ciliated photoreceptors)	Distal retina (ciliated photoreceptors)	Binding of RdCVF to a neuroplastin-like protein	Not found
*Branchiostoma lanceolatum* (Amphioxus)	*NXNLa*	Distal retina (ciliated photoreceptors)	Distal retina (ciliated photoreceptors)	Binding of RdCVFa to a neuroplastin-like protein	Found. Broadly expressed
	*NXNLb*	Expression very low	Expression very low		
	*NXNLc*				
	*NXNLd*				
*Petromyzon marinus* (Lamprey)	*NXNL*	Rods and cones	Rods	Binding of RdCVFa to BSG1 expressed by the retina	Retinal pigment epithelium
*Mus musculus*	*NXNL1*	Rods and cones	Rods	Binding of RdCVFa to BSG1 expressed by the retina	Retinal pigment epithelium
	*NXNL2*	Cells of the *area postrema*	Cells of the *area postrema*	Not found	

## Data Availability

The original contributions presented in the study are included in the article/supplementary material, further inquiries can be directed to the corresponding author.
